# The Acute Physiological Responses of Eccentric Cycling During the Recovery Periods of a High Intensity Concentric Cycling Interval Session

**DOI:** 10.3389/fphys.2020.00336

**Published:** 2020-04-17

**Authors:** Amelia J. Harrison, Catriona A. Burdon, Herbert Groeller, Gregory E. Peoples

**Affiliations:** ^1^Discipline of Medical and Exercise Science, School of Medicine, University of Wollongong, Wollongong, NSW, Australia; ^2^Centre for Medical and Exercise Physiology, School of Medicine, University of Wollongong, Wollongong, NSW, Australia; ^3^Graduate Medicine, School of Medicine, University of Wollongong, Wollongong, NSW, Australia

**Keywords:** high intensity interval training, eccentric cycling, recovery period, concurrent training, oxygen consumption, HIIT

## Abstract

Eccentric and concentric exercise is associated with disparate acute and chronic responses. We uniquely interspersed workload equivalent eccentric cycling during each recovery period of a high intensity interval training (HIIT) cycling trial to determine acute cardiopulmonary, thermal and psycho-physiological responses. Twelve males [age 28 years (SD 6), peak oxygen consumption 48 mL ⋅ kg^–1^ ⋅ min^–1^ (SD 6)] completed two high intensity interval cycling trials [4 × 5 min, 60% peak power output (PPO)] separated by 7–10 days. The **CON_R_** trial required participants to cycle concentrically during each recovery period (5 min, 30% PPO). The **ECC**_R_ trial modified the recovery to be eccentric cycling (5 min, 60% PPO). High intensity workload (**CON_R_**: 187 ± 17; **ECC**_R_: 187 ± 21 W), oxygen consumption (**CON_R_**: 2.55 ± 0.17; **ECC**_R_: 2.68 ± 0.20 L ⋅ min^–1^), heart rate (**CON_R_**: 165 ± 7; **ECC**_R_: 171 ± 10 beats ⋅ min^–1^) and RPE legs (**CON_R_**: 15 ± 3; **ECC**_R_: 15 ± 3) were equivalent between trials. Eccentric cycling recovery significantly increased external workload (**CON_R_**: 93 ± 18; **ECC**_R_: 196 ± 24 W, *P* < 0.01) yet lowered oxygen consumption (**CON_R_**: 1.51 ± 0.18; **ECC**_R_: 1.20 ± 0.20 L ⋅ min^–1^, *P* < 0.05) while heart rate (**CON_R_**: 132 ± 13; **ECC**_R_: 137 ± 12 beats ⋅ min^–1^) and RPE of the legs (**CON_R_**: 11 ± 7; **ECC**_R_: 12 ± 7) remained equivalent. There was no significant difference in the aural temperature between the trials (**ECC**_R_: 37.3 ± 0.1°C; **CON_R_**: 37.4 ± 0.1°C, *P* > 0.05), yet during recovery periods mean skin temperature was significantly elevated in the **ECC**_R_ (**ECC**_R_: 33.9 ± 0.2°C; **CON_R_**: 33.3 ± 0.2°C, *P* < 0.05). Participants preferred **ECC**_R_ (10/12) and rated the **ECC**_R_ as more achievable (82.8 ± 11.4 mm) than **CON_R_** (79.4 ± 15.9 mm, *P* < 0.01). In conclusion, eccentric cycling during the recovery period of a HIIT training session, offers a novel approach to concurrent training methodology. The unique cardiopulmonary and skeletal muscle responses facilitate the achievement of both training stimuli within a single exercise bout.

## Introduction

Intervals of high intensity exercise, interspersed with periods of low physical demand (recovery) increase the capacity to perform external work, when compared to equivalent moderate continuous exercise (Åstrand et al., 1960; Fox et al., 1969). Despite these lowered training volumes and time spent exercising, high intensity interval training (HIIT) is effective in eliciting significant cardiopulmonary, metabolic and musculoskeletal remodeling similar to that observed in continuous endurance training (Gibala et al., 2006; Burgomaster et al., 2008; Hafstad et al., 2011; MacInnis and Gibala, 2017). Furthermore, the adaptive utility of HIIT is clearly demonstrated across a range of physical work capacities and disease states (Tjønna et al., 2008; Gibala et al., 2012; Weston et al., 2014). These characteristics have led some investigators to propose the adoption of HIIT as a training strategy to overcome commonly cited barriers to exercise participation such as insufficient time and reduced motivation (Reichert et al., 2007; Gibala et al., 2015).

While the HIIT has been the primary focus, with respect to manipulation of exercise duration and intensity, the recovery period between each interval of work has received comparatively less attention (Fox et al., 1969). As such, the current investigation sought to deliberately manipulate the recovery period of a single HIIT session by modifying the contractile requirement of the skeletal muscle to apply an eccentric load. Eccentric cycling uncouples external work from metabolic and cardiovascular demand (Dufour et al., 2004; Lewis et al., 2018), and so we first hypothesized that twofold more eccentric external work (eccentric cycling) would not impose a physiological burden significantly different to the usual concentric (positive work) recovery period during a single HIIT session.

Eccentric work, as demonstrated by eccentric cycling, is characterized by unique motor processing, reduced motor unit recruitment, preferential activation of higher threshold fibers and a significantly lower metabolic demand compared to positive work (Dufour et al., 2007; Herzog, 2014; Duchateau and Enoka, 2016; Lewis et al., 2018). It is this latter attribute, that has received most research interest, where for a given concentric work load, eccentric work can be up to four and fivefold higher for cycling and treadmill exercise, respectively, and still elicit an equivalent cardiopulmonary and metabolic response (Davies and Barnes, 1972; Dufour et al., 2004). Given eccentric exercise requires work to be performed on the muscle rather than by the muscle as observed in concentric muscle activations (Dean, 1988), we also questioned what the thermoregulatory consequences were for the additional workload undertaken during the HIIT eccentric recovery periods.

During eccentric exercise the potential energy supplied by the treadmill or cycle ergometer is lost via the lengthening muscle and released primarily as heat (Davies and Barnes, 1972). Indeed, heat production during eccentric work will exceed metabolic energy liberation by a factor of three (Nielsen, 1966). The physiological implications of this additional thermal load is an increase in cutaneous blood flow and sweating compared to muscle shortening exercise in order to maintain thermal equilibrium (Nielsen, 1966; Nielsen et al., 1972). Therefore, our second hypothesis was that eccentric work, performed during HIIT recovery periods as eccentric cycling, would significantly increase body temperature, measured at the core and as the mean across the skin, compared to positive work (concentric cycling recovery).

The collective aim of this study was therefore to determine the cardiopulmonary, metabolic and thermoregulatory effects of replacing concentric cycling with eccentric cycling during the recovery periods of a single HIIT cycling session. We hypothesized that the additional overall external work achieved by eccentric cycling during recovery periods would not perturbate cardiopulmonary or metabolic responses but may result in an increased thermal load.

## Materials and Methods

### Participants

Twelve (*n* = 12) male participants [age 28 years [standard deviation (SD) 6], mass 78 kg (SD 13), stature 1.80 m (SD 0.09), peak oxygen consumption 48 mL ⋅ kg^–1^ ⋅min^–1^ (SD 6)], each regularly engaging in moderate-to-vigorous intensity physical activity per week, completed medical screening questionnaires and provided voluntary written informed consent. All procedures were approved by the University of Wollongong Human Ethics Research Committee (2018/059).

### Experimental Design

**FIGURE 1:**
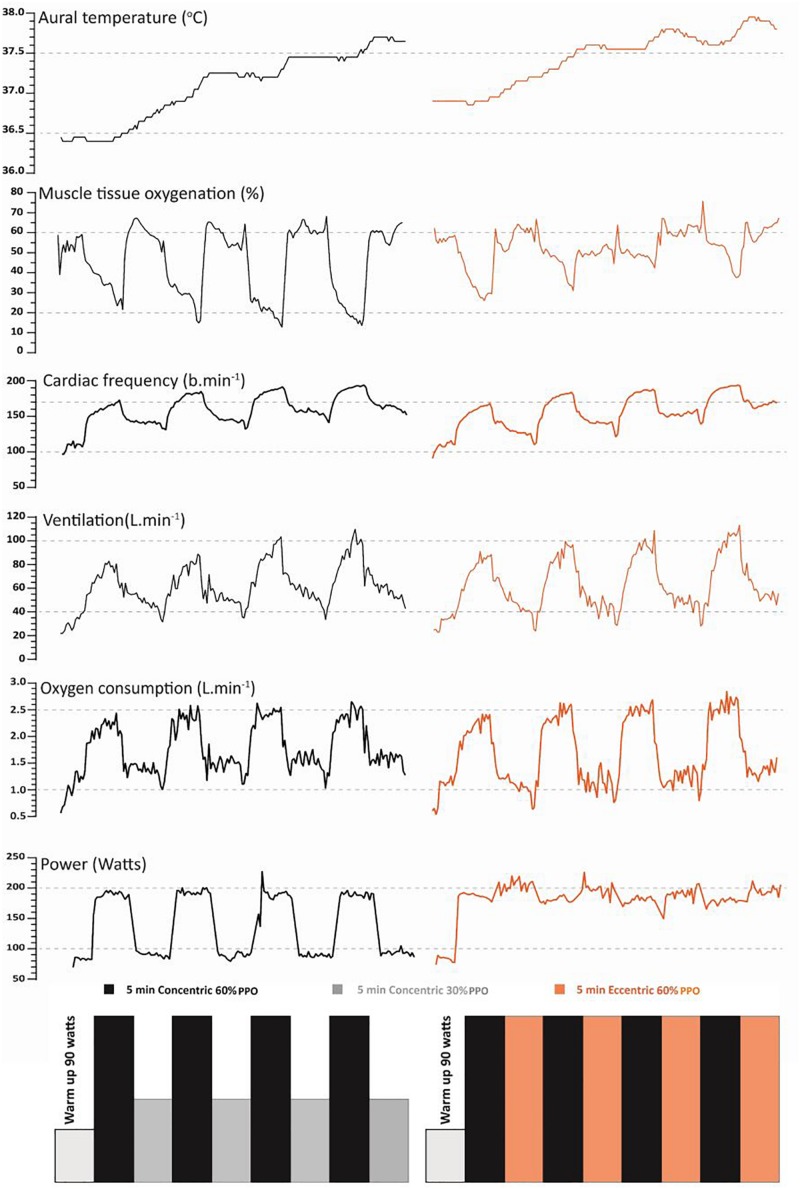
Representative traces of the typical aural temperature (°C), muscle tissue oxygenation (%), heart rate (beats ⋅ min^–1^), minute ventilation (L ⋅ min^–1^), oxygen consumption (L ⋅ min^–1^) and power output (W) from an individual participant in response to the high intensity (60% PPO) interval (4 × 5 min) recumbent cycling trials that differed according to the 5 min recovery periods [concentric cycling at 30% PPO (**CON**_R_) and eccentric cycling at 60% PPO (**ECC**_R_)].

Participants visited the laboratory on four occasions to; (i) determine peak aerobic power, (ii) familiarize participants to semi-recumbent eccentric cycling, and (iii, iv) complete two high intensity interval trials; separated by 7–10 days, that were randomized and conducted in balanced order. One of the trials (**CON_R_**) required participants to perform 4 × 5-min work intervals at 60% of the upright cycling peak power output (PPO). Each work interval was followed by a 5-min recovery period at a 30% PPO. In the alternate trial (**ECC**_R_) only the recovery period was modified with participants performing eccentric cycling at 60% of the upright cycling PPO. Thus, in **ECC**_R_, participants maintained a workload equivalent to 60% PPO for 40 min with only the mode of cycling changing between concentric (work interval) and eccentric (recovery period) cycling (Figure 1). Prior to attending the laboratory, participants were asked to consume at least 140 g of carbohydrate, and refrain from atypical strenuous physical activity, alcohol and caffeine in the 24 h prior to each of the experimental trials. All assessments were conducted in a climate-controlled laboratory set at 22°C and 30% relative humidity.

### Peak Aerobic Power

Body mass and stature was recorded upon arrival at the laboratory. Participants then completed a 5 min cycling warmup prior to commencing the incremental ramp protocol. The cycle ergometer (Excalibur sport, Lode, Groningen, Netherlands) was adjusted for each participant prior to commencing the incremental ramp protocol; cycling at 90 W and then 120 W for 2 min, thereafter workload increased by 1 W ⋅2 s^–1^ until volitional termination corresponding to the PPO. Expired gases were collected using a two-way breathing valve (Hans Rudolph, Shawnee, OH, United States) and analyzed using a calibrated gas analysis system (TrueOne 2400, Parvo Medics, East Sandy, UT, United States). The highest oxygen consumption over a 30-s period was recorded as the peak (L ⋅ min^–1^). Heart rate was obtained continuously (800cx, Polar, Finland) during the protocol.

### Recumbent and Eccentric Cycling Familiarization

On the second visit participants performed eccentric cycling on a customized recumbent ergometer (Lewis et al., 2018), until they could consistently maintain 5 min of eccentric cycling at 60% (±5 W) of their peak workload in a well-coordinated manner. The ergometer was retrofitted with a 240 V, 0.75 kW asynchronous electric motor (MasterDrive Simovert Vector Control, Siemens, Erlangen, Germany) to facilitate eccentric cycling. Eccentric workload was measured using instrumented (0–4000 W ± 0.5%) bicycle cranks (Science Road Power Meter, SRM, Jülich, Germany) that allowed power (W) and cadence (rev ⋅ min^–1^) to be recorded (1 Hz) using an wireless display unit (Edge 520, Garmin, Kansas, KS, United States).

### HIIT Exercise Trials

Participants arrived at the laboratory to perform, in balanced order, 4 × 5 min concentric cycling (60% PPO) (Figure 1). This relative intensity of PPO was chosen, as it is known to elicit near maximal cardiopulmonary responses (i.e., >80% peak heart rate) yet achievable for minutes. The recovery periods (4 × 5 min) were performed cycling either concentrically at 30% of the PPO (**CON**_R_) or eccentrically at 60% of the PPO (**ECC**_R_) on the custom-built recumbent ergometer.

Participants were instrumented with a heart rate monitor, thermistors (skin and auditory canal), near infrared spectroscopy (NIRS), pulse oximeter and oronasal mask and then rested seated on the ergometer for 5 min prior to commencing either a **CON**_R_ or **ECC**_R_ 40 min exercise trial.

### Experimental Measures

Expired gases were measured continuously and then averaged over a 15-s period for volume, carbon dioxide and oxygen concentration using an open circuit gas-analyzer (TrueOne 2400, Parvo Medics, East Sandy, UT, United States). Prior to each use, the flow meter and gas analyzers were calibrated using a 3-L volumetric syringe and a two-point alpha standard gas calibration, respectively. Heart rate was obtained continuously using ventricular depolarization (800cx, Polar, Finland). Muscle tissue oxygenation was recorded continuously (2 Hz) to the nearest percentile on the anterior surface of the thigh (*vastus lateralis*), mid-way between the anterior superior iliac spine and the patella (90° hip and knee flexion) using a NIRS device (Moxy Monitor, Fortiori Design, Minnesota, MN, United States). Arterial oxygen saturation was measured using a pulse oximeter (Nellcor Bedside SpO_2_ Patient Monitoring System, PM100N, Medtronic, United States) attached to the index finger of the right hand. Core and body skin temperatures were also estimated using aural (Edale instruments Ltd., Cambridge, United Kingdom), and skin (YSI type-EU, Yellow Springs Instruments, Yellow Springs, OH, United States) thermistors, respectively. Aural temperature was recorded using an ear-molded plug that was passively insulated (cotton wool) to shield the thermistor from the thermal environment. Skin thermistors measured temperature from eight sites (forehead, right chest, right scapula, right upper arm, right forearm, right dorsal hand, right anterior thigh, and right calf) that were used to calculate an area-weighted summation of mean skin temperature (ISO 9886, 2004). All temperatures were sampled continuously (15-s intervals; 1206 Series Squirrel, Grant Instruments Ltd., Shepreth, Cambridgeshire, United Kingdom). Thermistors were calibrated against a certified reference thermometer (Dobros total immersion, Dobbie Instruments, Sydney, NSW, Australia) using a stirred water bath, and across physiologically relevant temperatures. The average value for the experimental measures in the last 90 s of each high intensity work interval and recovery period (5 min, respectively) was used for statistical analysis.

Psychophysical responses during and after interval exercise were also assessed. Ratings of perceived exertion for whole body, chest and legs were recorded using the 15-point Borg scale (Borg, 1982) in the final 90 s of each high intensity work interval and recovery period, respectively. Using a 100-mm visual analogue scale (VAS), participants rated their perception of delayed onset of muscle soreness (DOMS) of the lower limbs with 0 mm = *no pain* and 100 mm = *unbearable pain* at 1, 24, 48, and 72 h post trial. All DOMS VAS ratings occurred while the participants were at 90° hip and knee flexion against a wall (a wall sit). To assess the potential for translation to practice, the trial achievability (not achievable – extremely achievable) was assessed using a 100 mm VAS scale. Participants were asked, 10 min after the completion of the trial, to place a pen mark on the scale to “rate the trial in terms of achievability?” Equally, once both trials were completed, participants were simply asked to select which trial was their preferred exercise training session.

### Statistical Analysis

This experiment followed a randomized, latin square within-subjects study design as each participant performed **CON**_R_ and **ECC**_R_. Two-way repeated measures analyses of variance (two conditions and 10 time points) were used to identify any differences in workload (power output), physiological (oxygen consumption, heart rate, muscle tissue oxygenation, arterial oxygen saturation, and body temperature), and subjective responses (perceived exertion and delayed onset muscle soreness) between the concentric and eccentric recovery periods during the two trials. A student’s *t*-test (two-tailed) was used to identify any differences in body mass, sweat rate, temperature at rest, change in temperature, and post-exercise subjective responses. A Tukey’s *post hoc* analysis, allowing for multiple comparisons was utilized to determine statistical significance which was set at alpha < 0.05. Data was checked for normal distribution using the Shapiro–Wilk test. Data is presented as mean values and standard error of the mean for *n* = 12, unless otherwise stated. A sample size of four (*n* = 4) was determined (using GPower 3.1.9.4) to be sufficient to demonstrate a difference (power: 0.95, alpha: 0.05; effect size dz; 6.0) between two dependent means (SD) (within subjects) for external work during recovery (concentric versus twofold elevated eccentric – one tail) where oxygen consumption was estimated to be equivalent between conditions [<1 L/min according to Dufour et al. (2004)].

## Results

### Peak Aerobic Power and Familiarization

At peak oxygen consumption [absolute: 3.75 L ⋅ min^–1^ (SD 0.60), relative: 48 mL ⋅ kg^–1^ ⋅min^–1^ (SD 6)] the mean peak heart rate and PPO were 185 beats ⋅ min^–1^ (SD 15) and 333 W (SD 43), respectively, confirming the subjects were aerobically conditioned. During the familiarization session, participants completed two 5 min blocks of eccentric cycling at 60% PPO, and during the second block power output remained ± 12% around the mean.

### High Intensity Work Periods

**FIGURE 2:**
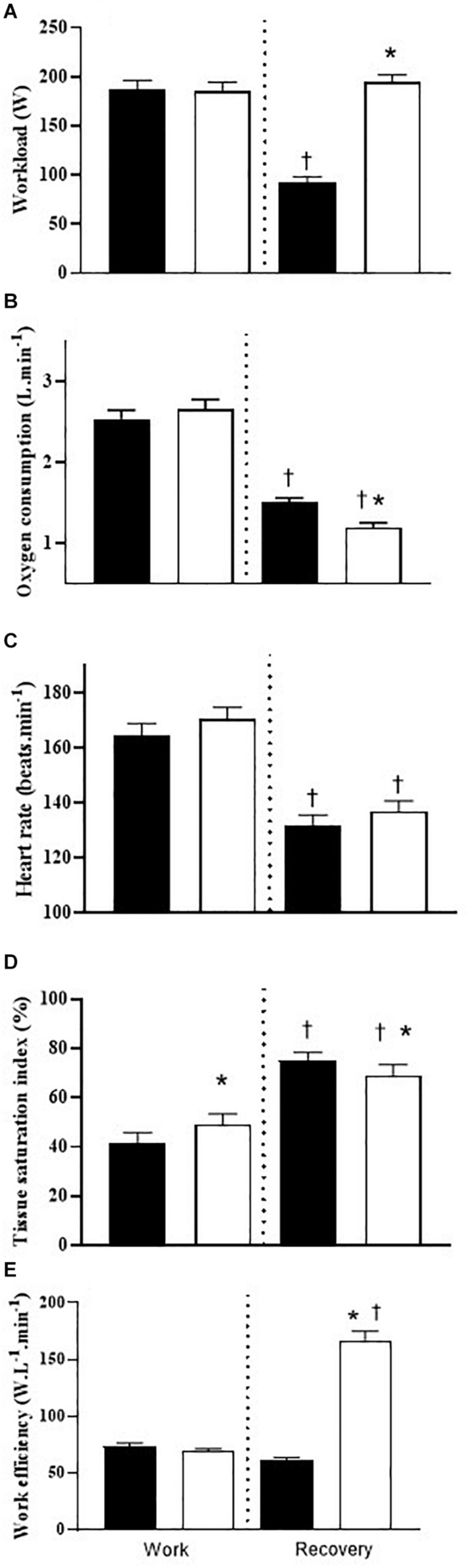
**(A)** Mean workload (watts) (*n* = 12); **(B)** oxygen consumption (L ⋅ min^–1^) (*n* = 12); **(C)** heart rate (beats ⋅ min^–1^) (*n* = 11); **(D)** muscle tissue oxygenation [tissue saturation index (%)] (*n* = 10); and **(E)** work efficiency (*W* ⋅ L^–1^ ⋅ min^–1^) (*n* = 12) during work and recovery intervals pertaining to the **CON**_R_ trial (black filled) and **ECC**_R_ trial (white filled). *P < 0.05 between **CON**_R_ versus **ECC**_R_ within either work or recovery periods. ^†^*P* < 0.05 between work and recovery within either **CON**_R_ or **ECC**_R_ trials. Data is expressed as mean ± SEM.

The mean workload across the four intervals of high intensity concentric cycling were equal for both **CON**_R_ (187 ± 5 W) and **ECC**_R_ trials (186 ± 5 W, *P* > 0.05) (Figure 2A). As such, there was no significant difference in mean oxygen consumption (**CON**_R_: 2.55 ± 0.05; **ECC**_R_: 2.68 ± 0.06 L ⋅ min^–1^, *P* > 0.05) (Figure 2B), heart rate (**CON**_R_: 165 ± 2; **ECC**_R_: 171 ± 3 beats ⋅ min^–1^, *P* > 0.05) (Figure 2C) or arterial oxygen saturation (**CON**_R_: 95 ± 1%; **ECC**_R_: 94 ± 1%, *P* > 0.05) between trials. As a result, the mean relative external work (efficiency) was equivalent between the two trials (**CON**_R_: 74 ± 1; **ECC**_R_: 70 ± 1 W ⋅ L^–1^ ⋅ min^–1^, *P* > 0.05) (Figure 2E). In both trials, muscle tissue oxygenation was reduced from the baseline (**CON**_R_: 72 ± 3%; **ECC**_R_: 73 ± 2%), as a result of the high intensity work (*P* < 0.05) and this reduction was lower in the **CON**_R_ trial (41 ± 3%) compared to the **ECC**_R_ trial (49 ± 2%, *P* < 0.05) (Figure 2D). Whole body rating of perceived exertion was equivalent between trials (**CON**_R_: 14 ± 0; **ECC**_R_: 14 ± 0; “Somewhat hard,” *P* > 0.05) and this was also evident for chest (**CON**_R_: 13 ± 0; **ECC**_R_: 13 ± 0; “Somewhat hard,” *P* > 0.05) and legs (**CON**_R_: 15 ± 1; **ECC**_R_: 15 ± 1; “Hard,” *P* > 0.05).

### Recovery Periods

The mean workload was significantly higher across eccentric recovery periods (**ECC**_R_: 196 ± 3 W) compared to concentric cycling recovery (**CON**_R_: 93 ± 3 W, *P* < 0.05) (Figure 2A). In spite of this, the mean oxygen consumption during recovery was significantly reduced from the exercise bout in both trials and this time the response was significantly lower during eccentric compared to concentric recovery (**CON**_R_: 1.51 ± 0.03; **ECC**_R_: 1.20 ± 0.03 L ⋅ min^–1^, *P* < 0.05) (Figure 2B). As a result, the mean relative external work (normalized to 1 L oxygen consumption) performed during the eccentric cycling recovery periods (**ECC**_R_: 168 ± 4 W ⋅ L^–1^ ⋅ min^–1^) was significantly higher compared to the concentric cycling recovery (**CON**_R_: 61 ± 1 W ⋅ L^–1^ ⋅ min^–1^, *P* < 0.05) (Figure 2E). Heart rate also decreased during cycling recovery and this response was equivalent between the **CON**_R_ and **ECC**_R_ trials (**CON**_R_: 132 ± 2 beats ⋅ min^–1^; **ECC**_R_: 137 ± 2 beats ⋅ min^–1^, *P* > 0.05) (Figure 2C). Muscle tissue oxygenation increased toward resting conditions and this response was augmented in the concentric (75 ± 2%) compared to the eccentric recovery periods (69 ± 2%, *P* < 0.05) (Figure 2D). The arterial oxygen saturation remained undisturbed during recovery periods (**CON**_R_: 95 ± 1%; **ECC**_R_: 94 ± 1%, *P* > 0.05). Rating of perceived exertion for whole body (**CON**_R_: 10 ± 0; **ECC**_R_: 11 ± 0, *P* > 0.05), chest (**CON**_R_: 10 ± 0; **ECC**_R_: 10 ± 0, *P* > 0.05) and legs (**CON**_R_: 11 ± 0; **ECC**_R_: 12 ± 0, *P* > 0.05) were equivalent between concentric and eccentric recovery periods.

### Body Temperature and Mass

**FIGURE 3:**
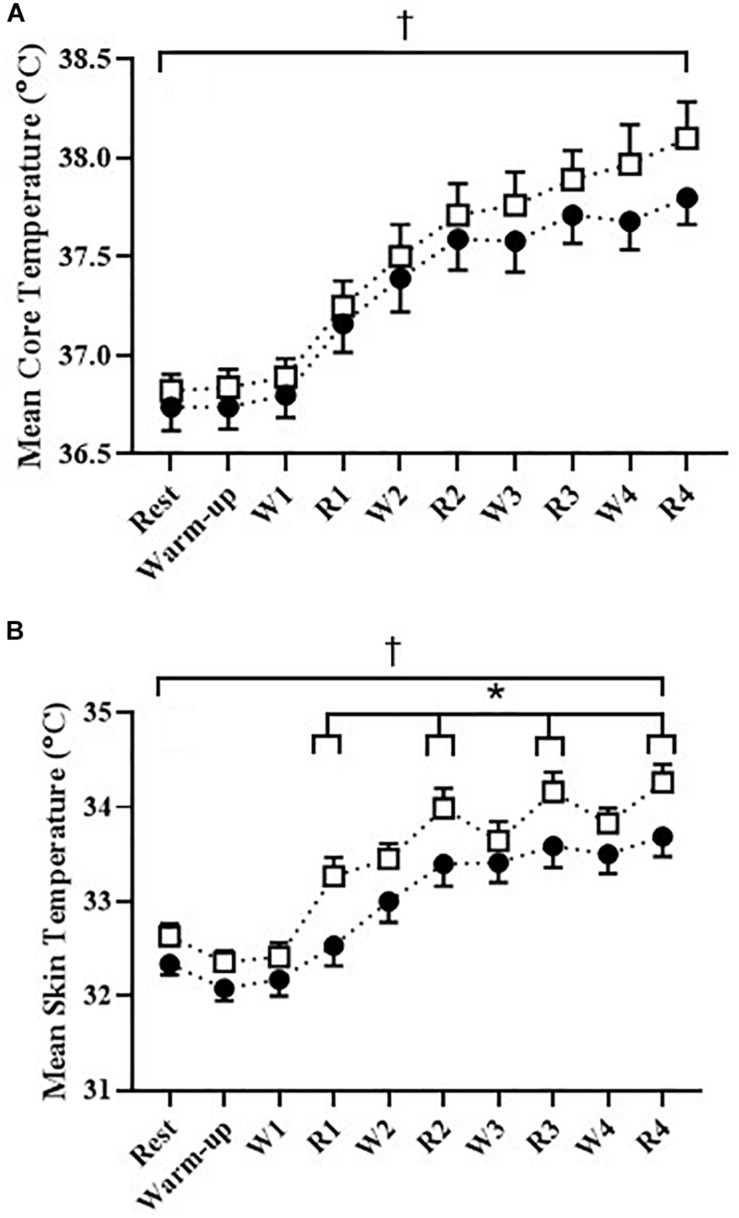
**(A)** Core body temperature (°C) (*n* = 10) and **(B)** mean skin temperature (°C) (*n* = 10) for **CON**_R_ (∙) and **ECC**_R_ (□) trials during seated rest, warm-up (60 watts), high intensity interval work (60% PPO, W1–W4) and recovery periods (**CON**_R_ 30%, **ECC**_R_ 60% PPO, R1–R4). *P < 0.05 **ECC**_R_ recovery versus **CON**_R_ recovery periods. ^†^*P* < 0.05 rest versus end trial (R4). Data is expressed as mean ± SEM.

**FIGURE 4:**
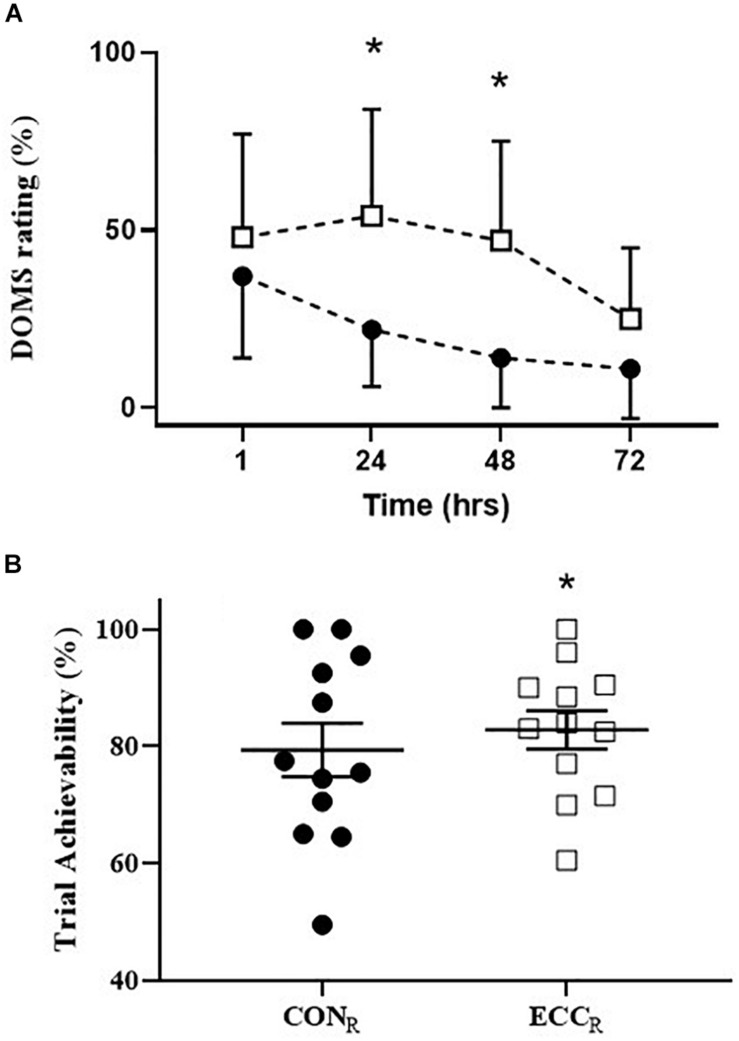
**(A)** Delayed onset of muscle soreness (%) (*n* = 10) following **CON**_R_ (∙) and **ECC**_R_ (□) trials at time points 1, 24, 48, and 72 h post exercise. *P < 0.05 **ECC**_R_ versus **CON**_R_ at time points 24 and 48 h post exercise. Data is expressed as mean ± SEM **(B)** trial achievability (%) (*n* = 12) for **CON**_R_ (∙) and **ECC**_R_ (□) as reported by the participants following the completion of each trial. *P < 0.05 **ECC**_R_ versus **CON**_R_. Data is expressed as individual data points with mean ± SEM.

Body core temperature (Figure 3A) and mean skin temperature (Figure 3B) significantly increased in both **CON**_R_ and **ECC**_R_ trials (*P* < 0.05, Figure 3). There was no significant difference in the body core temperature between the trials (*P* > 0.05). However, the mean skin temperature was significantly elevated in the **ECC**_R_ compared to **CON**_R_ trial during the recovery periods (*P* < 0.05, Figure 4B). Overall, the calculated sweat rate was greater in **ECC**_R_ (0.96 L ⋅ h^–1^ ± 0.06) compared to **CON**_R_ trials (0.83 L ⋅ h^–1^ ± 0.05, *P* < 0.05). As a result, body mass change was significantly greater in **ECC**_R_ (−0.69 ± 0.15 kg) compared to **CON**_R_ (−0.60 ± 0.11 kg, *P* < 0.05).

### Delayed Onset of Muscle Soreness

Eccentric cycling recovery periods resulted in increased muscle soreness of quadriceps at 24 and 48 h post-exercise (**CON**_R_ 24 h: 22 ± 5; 48 h: 14 ± 5%; **ECC**_R_ 24 h: 54 ± 9; 48 h: 47 ± 9%, *P* < 0.05) (Figure 4A).

### Trial Achievability

Subjects rated the **ECC**_R_ trial as significantly more achievable (82.8 ± 11.4 mm) than **CON**_R_ trial (79.4 ± 15.9 mm, *P* < 0.01) (Figure 4B) on the 100 mm VAS. Similarly, trial preference was higher for **ECC**_R_ (*n* = 10) than **CON**_R_ (*n* = 2).

## Discussion

This research explored the acute physiological response from two powerful adaptive stimuli, high intensity interval exercise and eccentric cycling, that were repetitively applied concurrently and in series during a single training session. Uniquely, we delivered the eccentric exercise, via eccentric cycling, within the recovery periods (**ECC_R,_** 4 × 5 min) of a routine, high intensity, recumbent cycling interval (4 × 5 min, 60% PPO) training session, which is known to elicit predictable physiological strain required to achieve HIIT. This unique cycling protocol has demonstrated several novel findings. First, the addition of 33% more external work in the **ECC**_R_ trial did not impose added cardiopulmonary, metabolic or psycho-physical strain during the high intensity work intervals compared to **CON**_R_ trial. In fact, during the recovery periods oxygen consumption was significantly lower in **ECC**_R_ trial**.** Second, while core temperature was similar between the two trials, mean skin temperature was significantly higher in **ECC**_R_ trial when the eccentric cycling recovery took place. Finally, the participants in this investigation clearly reported a preference for adopting eccentric cycling, delivering the higher external workload, over concentric cycling during the recovery periods of the high intensity interval training session, and thus validates the acceptability of this novel cycling protocol.

This investigation raises a physiological conundrum with respect to the definitions used for interval and concurrent training. The participants were exposed to four bouts of high intensity intervals that required ∼90 and ∼70% of peak heart and oxygen consumption, respectively, during these work intervals (Weston et al., 2014; MacInnis and Gibala, 2017). However, our methodology diverged significantly from convention with respect to the recovery period. Evidence shows the period of recovery after each work interval is normally characterized by a significant reduction in work (Fox et al., 1973). Such reduction in concentric workload optimizes blood flow and metabolic recovery and therefore ensures maintenance of sufficient intensity for the subsequent work interval (Buchheit and Laursen, 2013). In contrast, by utilizing eccentric cycling, external work could now be maintained, albeit in muscle now lengthening, during the recovery period without compromising completion of each of the 5-min high intensity work intervals.

Eccentric exercise, is known to uncouple the relationship between the force producing capacity (Katz, 1939) and the metabolic and cardiopulmonary demand (Abbott et al., 1952; Peñailillo et al., 2017), with a four (eccentric exercise) to one (concentric exercise) ratio observed (Dufour et al., 2004). Notwithstanding this relationship, we applied a conservative twofold difference in concentric and eccentric exercise work rate within this investigation, and our results confirm the uncoupling of external work and metabolic and cardiopulmonary responses even when immediately transitioning from high intensity cycling. In fact, completion of the eccentric recovery period, performed at a twofold higher workload (the conservative approach), required an oxygen consumption 10% lower (1.20 L ⋅ min^–1^) than the concentric recovery period (1.51 L ⋅ min^–1^, Figure 2B), with no difference observed in heart rate during that recovery. Additionally, our participants reported no difference in rating of perceived exertion (whole body, chest, and legs) between **ECC**_R_ and **CON**_R_, and is consistent with others (Henriksson et al., 1972). Importantly, when external work was normalized to oxygen consumption (Figure 2E) 61 W and 167 W were performed per liter of oxygen consumed during the concentric and eccentric exercise recovery periods, respectively. This 2.7-fold difference in work efficiency is lower than the 3–7-fold change observed by others (Abbott et al., 1952; Pahud et al., 1980; Dufour et al., 2004) and may relate to the absolute work rate not being fixed between the two conditions in our investigation. From a mechanistic viewpoint, Peñailillo et al. (2017), primarily attributed the reduction in oxygen cost of eccentric cycling to a lowered agonist and antagonist muscle activation to torque ratio.

During the **CON**_R_ recovery periods, muscle tissue oxygenation returned toward baseline, although, this was less pronounced during the recovery periods of **ECC**_R_. The difference in absolute work rate between **ECC**_R_ and **CON**_R_, transient changes in workload associated with interval training and the contrasting muscle tension, may partly explain this observation. Others have reported during continuous oxygen consumption-matched exercise, that there is no difference in muscle tissue oxygenation between eccentric and concentric cycling (Rakobowchuk et al., 2018). Furthermore, when external work is matched, an increased muscle tissue oxygenation (Muthalib et al., 2010; Peñailillo et al., 2017), faster oxygen consumption kinetics (Perrey et al., 2001) and reduced muscle activation (Peñailillo et al., 2017) are observed. Overall, the combined observations suggest that the overall reduction in oxygen cost of eccentric cycling is reflected by the state of the muscle, and for this current study, near recovery of this muscle oxygenation can occur within minutes when transitioning rapidly from concentric to eccentric contractions.

The most novel aspect of the current investigation centered upon the concurrent, and in series, stimuli that required the participant to transition rapidly between the work intervals and recovery periods, where the latter differed according to mode of contraction. In contrast, others have reported on the acute physiological responses of varying length eccentric cycling intervals where by a period of rest was provided as the recovery (Lipski et al., 2018). Physical training is often viewed in this way, as single-mode exercise, that consists of either endurance or strength/power activities (Coffey and Hawley, 2017). Classification of concurrent training is therefore endurance and strength/power-based activities typically performed within a single training session or across multiple training sessions (McCarthy et al., 2002; Coffey and Hawley, 2017). Concurrent training may elicit adaptive interference possibly due to differential molecular signaling or likely accumulated metabolic and neuromuscular fatigue as a consequence of increased training stress (Hickson, 1980). However, within the current investigation participants during **ECC**_R_ performed increased external work and therefore, were exposed to an elevation in mechanical stress but paradoxically, a reduced metabolic load within an acute bout of interval exercise.

The metabolic and molecular adaptive signaling associated with **ECC**_R_ has not previously been considered within the concurrent training literature. Exposure to a range of muscle actions has been advocated to optimize molecular and metabolic adaptations (Olsen et al., 2019). Receptor proteins tied to the extracellular matrix have been suggested to be sensitive to the tensile loading associated with eccentric muscle activation (Rindom and Vissing, 2016) where high mechanical loads are not necessarily a pre-requisite to elicit adaptation (Olsen et al., 2019). These findings are consistent with enhanced adaptations observed following eccentric cycling when compared to either concentric cycle exercise (LaStayo et al., 2000) or resistance training (LaStayo et al., 2003). Furthermore, eccentric exercise has both a different control strategy from the central nervous system and magnitude of muscle activation (Duchateau and Enoka, 2016; Peñailillo et al., 2017). Thus, it is possible to speculate whether the inclusion of eccentric cycling during the recovery period of a high intensity interval training session may induce unique physiological adaptations. What was clearly apparent from subjective responses of participants, when weight bearing for the first time after the interval session, is that **ECC**_R_ elicited considerably greater local muscle fatigue and reported levels of delayed onset muscle soreness. Yet, in an apparent contradiction, the participants reported both the **CON**_R_ and **ECC**_R_ as equivalently achievable, and in fact the **ECC**_R_ was statistically higher. Moreover, 10 of the participants also reported a preference to engaging in the training session that included an eccentric recovery period. Acknowledging that these self-reported survey questions were generated by the research group, the purpose was to simply capture the potential for translation to practice for this novel training session, and for this group, the inclusion of an eccentric recovery period was well accepted on face value. This opens up the possibility of a training study with a focus on adaptive responses to the concurrent stimulus. On the same theme, it was also self-reported by some participants (verbally, non-prompted) that at the commencement of each concentric work interval, that immediately followed an eccentric recovery period, a sensation of an enhanced ability to produce concentric torque. Acutely, residual force enhancement has been well documented following lengthening muscle activations (Abbott et al., 1952; Peñailillo et al., 2017). Nevertheless, when eccentric cycling is repeated over the course of 2 weeks, changes in muscle fascicle length and muscle soreness are reported to be reduced on the second occasion in combination with adaptations to the muscle tissue that may modify its contractile performance (Penailillo et al., 2015; Valladares-Ide et al., 2019). The mechanisms are not yet fully understood but may, in part explain, our participants’ preference to engaging in **ECC**_R_ over **CON**_R_.

Contrary to our initial hypothesis, **ECC**_R_ and **CON**_R_ both observed a 1.0–1.2°C increase in core temperature. Yet given the current responses we observed, had the **ECC**_R_ workload been increased to be normalized for oxygen consumption with **CON**_R_, discernible differences would likely have been detected (Pahud et al., 1980). Nevertheless, T_skin_ in **ECC**_R_ was significantly higher, with a markedly different profile evident in comparison to **CON**_R_ (Pahud et al., 1980; Knuttgen et al., 1982). **ECC**_R_ skin temperatures was highest with each eccentric cycling recovery period and then declined in the subsequent concentric work interval, thus taking on a distinctive sawtooth profile in the final intervals. A threefold increase in heat production, high muscle temperature and a twofold increase in cutaneous blood flow characterize thermoregulatory differences between negative (eccentric) and positive (concentric) work (Nielsen, 1966; Nielsen et al., 1972; Pahud et al., 1980), explaining the transient rises in skin temperature observed in the current study. Nonetheless, the thermoregulatory response to metabolically matched or workload equivalent eccentric cycling deserves increased scrutiny. This is particularly important given that the application for eccentric cycling has been rapidly adopted within exercise prescription, including special populations.

## Conclusion

This study has developed a novel cycling protocol, preferred by the participants, where the metabolic advantages of eccentric cycling were utilized during the recovery periods of a single training session to increase total external work by 33% while the cardiopulmonary and metabolic responses remained unperturbed and synonymous with HIIT. This unique approach to concurrent training, with the dual stimuli, opens up a range of new questions pertaining to central and peripheral adaptive responses to exercise training.

## Data Availability Statement

The datasets generated for this study are available on request to the corresponding author.

## Ethics Statement

The studies involving human participants were reviewed and approved by the Human Research Ethics Committee, University of Wollongong, Australia. The patients/participants provided their written informed consent to participate in this study.

## Author Contributions

HG, GP, and CB conceived and designed the research. AH and CB conducted the experiments. All authors analyzed the data, wrote the manuscript, and read and approved the manuscript.

## Conflict of Interest

The authors declare that the research was conducted in the absence of any commercial or financial relationships that could be construed as a potential conflict of interest.
